# jsPhyloSVG: A Javascript Library for Visualizing Interactive and Vector-Based Phylogenetic Trees on the Web

**DOI:** 10.1371/journal.pone.0012267

**Published:** 2010-08-18

**Authors:** Samuel A. Smits, Cleber C. Ouverney

**Affiliations:** Department of Biological Sciences, San José State University, San José, California, United States of America; BC Centre for Excellence in HIV/AIDS, Canada

## Abstract

**Background:**

Many software packages have been developed to address the need for generating phylogenetic trees intended for print. With an increased use of the web to disseminate scientific literature, there is a need for phylogenetic trees to be viewable across many types of devices and feature some of the interactive elements that are integral to the browsing experience. We propose a novel approach for publishing interactive phylogenetic trees.

**Methods/Principal Findings:**

We present a javascript library, jsPhyloSVG, which facilitates constructing interactive phylogenetic trees from raw Newick or phyloXML formats directly within the browser in Scalable Vector Graphics (SVG) format. It is designed to work across all major browsers and renders an alternative format for those browsers that do not support SVG. The library provides tools for building rectangular and circular phylograms with integrated charting. Interactive features may be integrated and made to respond to events such as clicks on any element of the tree, including labels.

**Conclusions/Significance:**

jsPhyloSVG is an open-source solution for rendering dynamic phylogenetic trees. It is capable of generating complex and interactive phylogenetic trees across all major browsers without the need for plugins. It is novel in supporting the ability to interpret the tree inference formats directly, exposing the underlying markup to data-mining services. The library source code, extensive documentation and live examples are freely accessible at www.jsphylosvg.com.

## Introduction

Constructing phylogenetic trees is a fundamental task in multiple fields of biology, including evolutionary, genetic, molecular and conservational biology. Many software packages [1]–[4] have been created to address the need for constructing trees as static images for use in printed literature. Online access to scientific literature has recently opened up new possibilities yet presents additional challenges, however. For instance, phylogenetic trees need to be presented at varying resolutions in order to be optimized for both large monitors and small mobile devices. Furthermore, the dynamic nature of the web demands interactive elements and rich analytics integrated within a tree.

We introduce a novel javascript library for phylogenetic visualizations, jsPhyloSVG, which addresses many of the fundamental challenges involved with viewing phylogenetic trees online, including: generating scalable, navigatable trees that render in all major browsers without the need for plugins, integrating complex charting and boasting interactive features.

## Methods

jsPhyloSVG is designed to be a flexible, lightweight javascript library for building interactive and complex phylogenetic trees in a web-based environment with the broadest range of accessibility. To this end, it renders Scalable Vector Graphics (SVG) files using client-side javascript. SVG offers a number of key features, including: the ability to pan and zoom without loss of resolution, interactive capabilities, extensibility with javascript and CSS, small file size and is supported by HTML5. SVG is supported out-of-the-box by all major browsers with the exception of Internet Explorer (version 9 is anticipated to support SVG). However, the software degrades gracefully – adjusts to inferior capabilities of the browser without producing an error message – within Internet Explorer and automatically generates an analogous format, Vector Markup Language (VML). The VML format displays an identical tree but is unable to support the interactive features that SVG is capable of.

Javascript rendering of phylogenetic trees also offers a number of advantages. Firstly, all modern browsers implement javascript natively. Alternative web-based approaches to rendering phylogenetic trees, such as implementations using Flash (iTOL [5]) or Java applets (PhyloWidget [6]), are limited by requiring plugins (see Table 1) that are not natively supported by many browsers and a broad range of mobile devices, including recent tablet devices. Secondly, although the javascript implementation utilizes the web browser for its Graphical User Interface (GUI), it does not require an internet connection. Other web-based implementations [7], [8] circumvent requiring plugins by generating the tree server-side, and therefore require online connectivity. Finally, the use of javascript enables one to harness the power of many popular javascript libraries, such as jQuery [9] and the Yahoo User Interface Library [10], and have the ability to create complex, interactive trees.

**Table 1:** Comparison of common, scalable and interactive phylogenetic tree viewers that are intended for the web.

	jsPhyloSVG	TreeVector	PhyloWidget	iTol
**User Interface (for generation)**	Web	Command-line	Web	Web
**Open source**	Yes	Yes	Yes	No
**Plugins required**	None	None	Java	Flash
**Supports Charting**	Yes	No	No	Yes
**Supports Newick and phyloXML**	Both	Newick	Newick	Both
**What Renders the Visualization**	Browser	Server	Browser	Server
**Rectangular and Circular Phylograms**	Both	Rectangular	Both	Both

It is important to note, that jsPhyloSVG is the only solution that is capable of rendering phylogenetic trees directly from the calculated inference within the browser without the need for plugins.

## Results and Discussion

### Core Functionality

**Figure 1:**
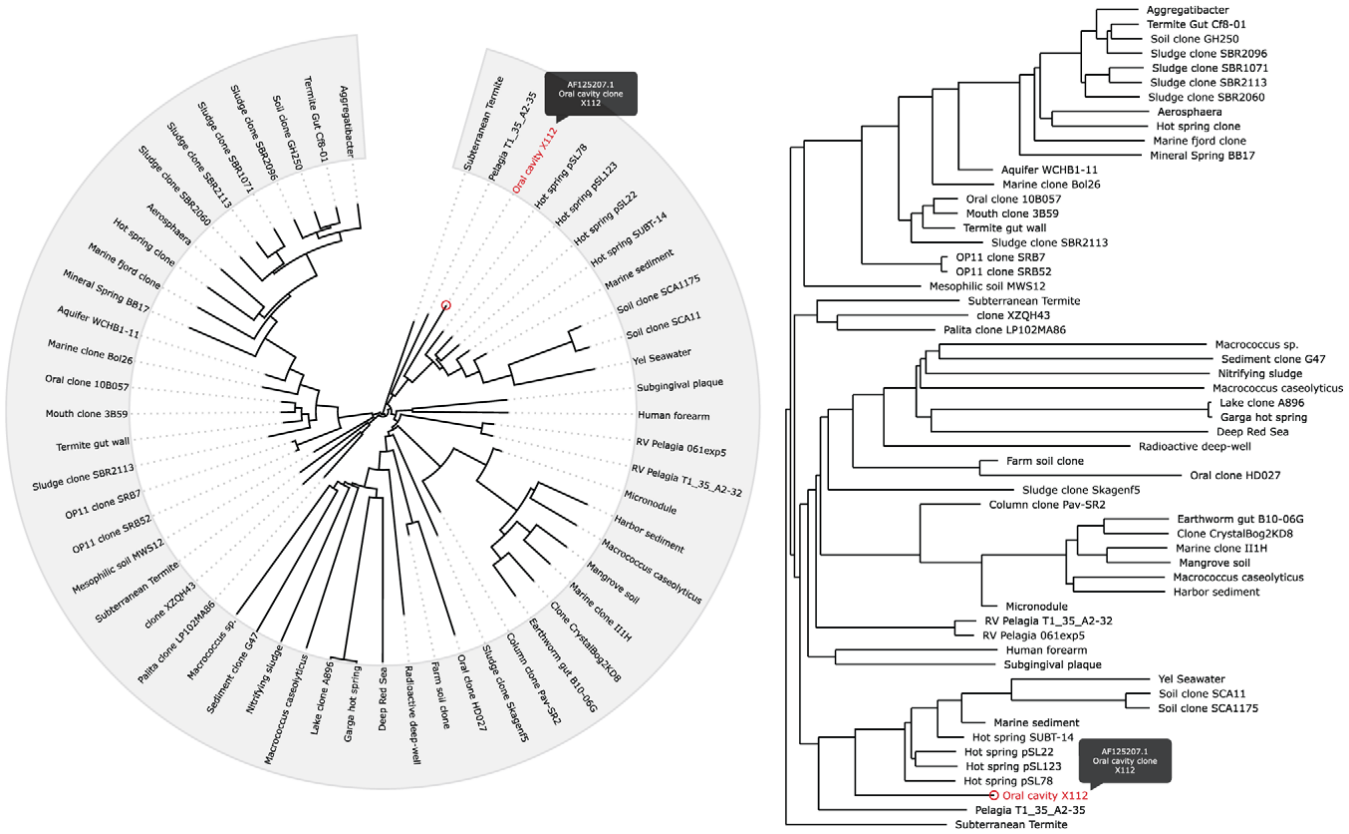
The two rendering formats: rectangular and circular phylograms. These tree formats are interchangeable and can be swapped between the two views dynamically. The figure illustrates customized behavior on mouse rollover. All events, including mouse rollovers, can be customized using javascript to perform any unique function within an application. The core functions of the library provide for mouse rollover and click events which by default may link to other websites or databases specified in the tree XML file.

The library renders both rectangular and circular phylograms from Newick and phyloXML [11] formats. Newick files (outputted by many popular phylogenetic packages, such as PHYLIP [1]) include at most only labels, edge lengths and bootstrap values; the tree renderings are therefore limited to these elements. Implementing the recently devised XML format specialized for phylogentic trees, phyloXML, offers the ability to include features such as text nodes that link to other websites or reveal descriptions on rollovers (see Figure 1). These core characteristics are present in both tree formats.

The javascript library consumes and interprets the raw Newick or phyloXML formats directly, from which trees are then generated. This approach is advantageous because it allows the readership to have access to the underlying tree source. Many online access journals and databases (PLoS [12], BioMed Central [13], Pubmed [14] and others) provide full papers in XML format while the phylogenetic trees themselves are provided as static images. It is of our opinion that the readership of online scientific literature would benefit greatly if the trees were also maintained in XML format. This would both provide greater transparency and allow the trees to be indexed by data-mining tools.

All of the stylistic qualities of the tree may be specified either programmatically as parameters or within the tree's XML file. Characteristics such as the font size or type, branch stroke width, mouse rollover events and scale can be easily customized. This offers the ability for each tree to be uniquely rendered using its predefined style in the XML file, or conversely allows for sites to override styles to maintain trees that are visually similar. Detailed explanations and documentation of available parameters and file formatting are available on our website.

### Charting

**Figure 2:**
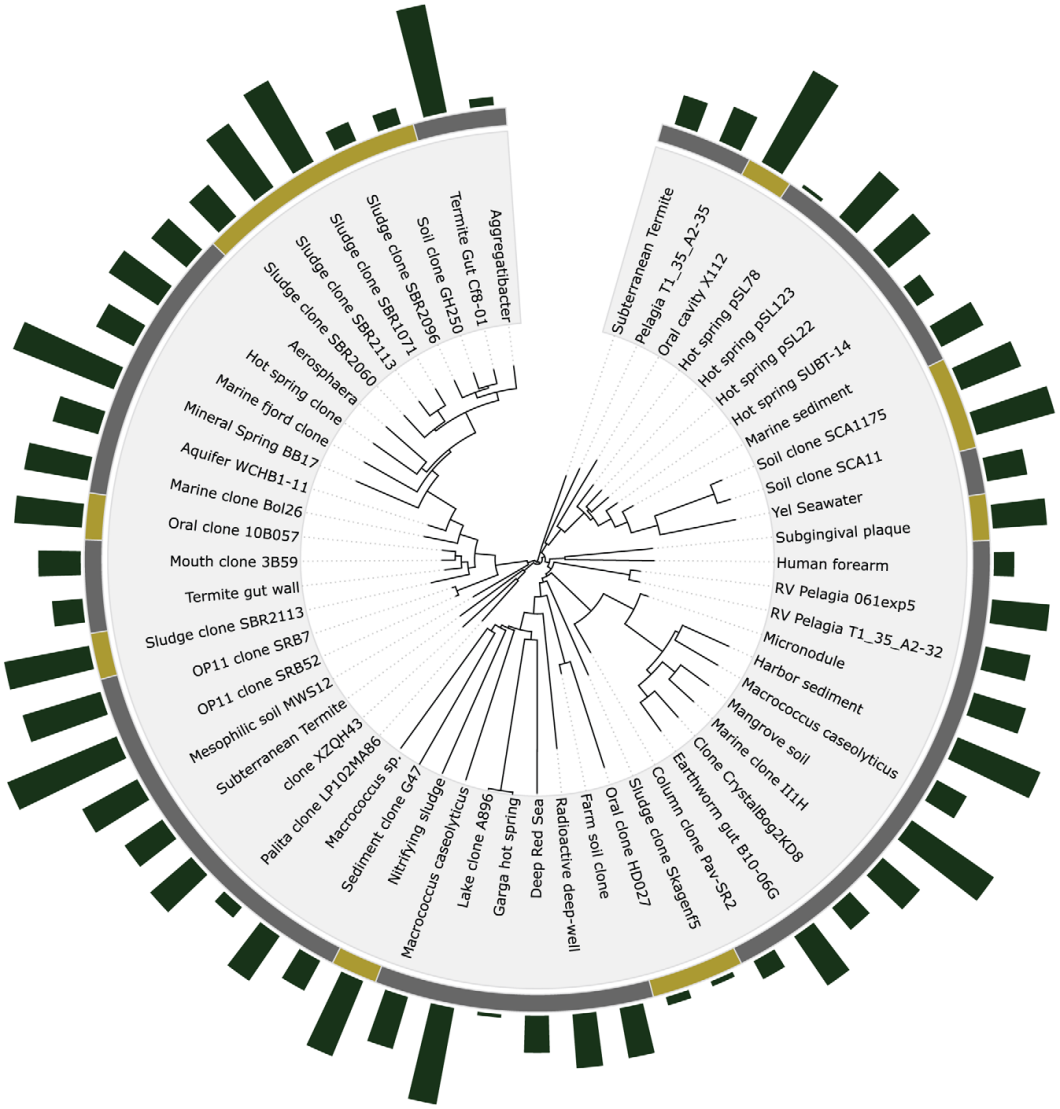
Integrated charting capabilities. Charting integrated within phylogenetic trees are powerful visualizations that are capable of conveying a lot of information. jsPhyloSVG currently supports two charting formats: binary and bar charts. Binary charts feature a ribbon that may be color-coded to indicate groups. Bar charts are utilized to convey a quantitative figure relative to others, such as the number of clones. An unlimited number of chart tracks may be integrated within the visualization.

The library integrates charting analytics within the tree visualization. Two charting formats are currently supported: binary and bar charts (see Figure 2). These reporting features are based on provided data within the tree XML file. The software normalizes the data and renders the charts according to user-defined styles in the XML file or parameters set at tree instantiation. jsPhyloSVG is flexible enough to an unlimited number of chart tracks to be added to the tree, with any number of stylistic characteristics.

### Limitations

The limitations of the library are primarily related to the authors' choice of using SVG to render the visualizations. Firstly, the format is not natively supported by current versions of Internet Explorer. This restriction has been overcome by automatically rendering the tree in the analogous format VML for these browsers. Secondly, SVG exposes its elements to the browser's Document Object Model (DOM) in order to allow for interactivity at an added slight computational cost. These computational tasks begin to significantly affect the performance of the browser, however, when rendering large trees that contain more than 2000 nodes (see Table S1). The choice to use SVG was deliberate in order to fulfill the primary objective of providing interactive capabilities despite the cost in performance for large trees.

### Conclusions

jsPhyloSVG is a comprehensive open-source solution for rendering dynamic phylogenetic trees. It leverages modern approaches by supporting HTML5 for the widest possible audience. It consumes phylogenetic inferences in Newick or XML formats and generates sophisticated trees that incorporate charting analytics in scalable vector images. The trees are also dynamic and provide instantaneous user feedback as they are navigated. Finally, the library is simple to extend and may be easily integrated into existing web applications.

### Availability and Requirements

Project home page: http://www.jsphylosvg.com

Operating System: Platform independent

Programming Language: Javascript

License: Open Source, GPL.

## Supporting Information

Table S1 Rendering performance across different browsers and devices. Number of seconds required to render SVG visualizations of phylogenetic trees with varying amounts of nodes across different browsers. Both Chrome and Firefox browsers were tested on a Lenovo Thinkpad X200 laptop (2.4 GHz, 4 Gb RAM) running Microsoft Windows 7. Safari was tested on a first generation Apple iPad.
(0.03 MB DOC)
