# Supplementary material for: jsPhyloSVG: A Javascript Library for Visualizing Interactive and Vector-Based Phylogenetic Trees on the Web
Source: PLoS One. 2010 Aug 18;5(8):e12267. doi: 10.1371/journal.pone.0012267 (PMC2923619; doi:10.1371/journal.pone.0012267)
Supplement: Table S1 — Rendering performance across different browsers and devices. Number of seconds required to render SVG visualizations of phylogenetic trees with varying amounts of nodes across different browsers. Both Chrome and Firefox browsers were tested on a Lenovo Thinkpad X200 laptop (2.4 GHz, 4 Gb RAM) running Microsoft Windows 7. Safari was tested on a first generation Apple iPad. (0.03 MB DOC) [file pone.0012267.s001.doc]

| **Nodes** | **Chrome 5.0 beta** | **Firefox 3.5** | **Safari (Apple iPad)** | |
| --- | --- | --- | --- | --- |
| **10** | 0.1 | 0.1 | 0.7 |  |
| **50** | 0.35 | 0.3 | 2.7 |  |
| **100** | 0.41 | 0.52 | 6.3 |  |
| **500** | 1.05 | 3.1 | 10.5 |  |
| **1000** | 1.9 | 6.4 | 22.8 |  |
| **2000** | 5.2 | 21.4 | 45.1 |  |
| **3000** | 11.9 |  |  |  |
| **4000** | 20.4 |  |  |  |
| **5000** | 32.3 |  |  |  |
